# Perception of ‘Back-Channeling’ Nonverbal Feedback in Musical Duo Improvisation

**DOI:** 10.1371/journal.pone.0130070

**Published:** 2015-06-18

**Authors:** Nikki Moran, Lauren V. Hadley, Maria Bader, Peter E. Keller

**Affiliations:** 1 Institute for Music in Human and Social Development (IMHSD), Reid School of Music, University of Edinburgh, Edinburgh, United Kingdom; 2 Psychology, School of Philosophy, Psychology and Language Sciences, University of Edinburgh, Edinburgh, United Kingdom; 3 Research Group: Music Cognition and Action, Max Planck Institute for Human Cognitive and Brain Sciences, Leipzig, Germany; 4 Music Cognition and Action Group, The MARCS Institute, University of Western Sydney, Penrith, Australia; Max Planck Institute for Human Cognitive and Brain Sciences, GERMANY

## Abstract

In witnessing face-to-face conversation, observers perceive authentic communication according to the social contingency of nonverbal feedback cues (‘back-channeling’) by non-speaking interactors. The current study investigated the generality of this function by focusing on nonverbal communication in musical improvisation. A perceptual experiment was conducted to test whether observers can reliably identify genuine versus fake (mismatched) duos from musicians’ nonverbal cues, and how this judgement is affected by observers’ musical background and rhythm perception skill. Twenty-four musicians were recruited to perform duo improvisations, which included solo episodes, in two styles: standard jazz (where rhythm is based on a regular pulse) or free improvisation (where rhythm is non-pulsed). The improvisations were recorded using a motion capture system to generate 16 ten-second point-light displays (with audio) of the soloist and the silent non-soloing musician (‘back-channeler’). Sixteen further displays were created by splicing soloists with back-channelers from different duos. Participants (N = 60) with various musical backgrounds were asked to rate the point-light displays as either real or fake. Results indicated that participants were sensitive to the real/fake distinction in the free improvisation condition independently of musical experience. Individual differences in rhythm perception skill did not account for performance in the free condition, but were positively correlated with accuracy in the standard jazz condition. These findings suggest that the perception of back-channeling in free improvisation is not dependent on music-specific skills but is a general ability. The findings invite further study of the links between interpersonal dynamics in conversation and musical interaction.

## Introduction

Effective communication during face-to-face interaction typically requires the accurate perception of nonverbal cues conveyed by body movements. During conversation, facial expressions, gestures, gaze and postural movements are used to reinforce, accentuate, or contradict the meaning of verbal utterances, and to regulate the dynamics of turn taking [1–3]. In the context of musical ensemble performance, body movements that accompany the production of musical sounds function similarly in communicating information about musical structure and expressive intentions to co-performers and audience members, as well as regulating the temporal coordination between performers [4–7]. The analogy with conversation is particularly apt in the case of improvised music, where co-performer interaction entails the spontaneous invention of musical material. The current study addresses the perception of nonverbal cues provided by the body movements of improvisers engaged in turn taking during musical performance. Our specific focus is on the role of ‘back-channeling’ cues.

The concept of communicative ‘back-channel’ refers to the idea that two simultaneous channels can be specified in an act of linguistic conversation: the speaker’s primary channel, and the addressee’s response, or ‘back channel’ [8]. Back-channel cues—incorporating vocalisations, facial expressions, gaze, and gestures—involve responsive feedback to the speaker to provide information about the addressee’s ongoing engagement in the dialogue. Evidence suggests that both such specific and general feedback cues are socially contingent within the dynamic process of face-to-face interaction, actively co-constituting the dialogue [3]. Moreover, observers can recognise contingency in dyads from body movements alone under variously stripped-back conditions, including silent video of interactors, and where animated representations are devoid of accompanying facial expression [9,10]. Observers are thus sensitive to social contingency in feedback cues associated with large-scale body movements produced in the context of interpersonal dialogue.

The current study investigated the generality of this ability by testing whether observers are sensitive to back-channeling cues provided by body movements in musical contexts. Dyadic–duo–musical interaction is a common situation in real life music-making. In the course of such ensemble performance, two musicians aim to integrate their contributions in such a way that audience members perceive a single performance event, rather than concurrent performances. One way in which musicians achieve this aim is through the use of nonverbal feedback to the performance of the other collaborating musicians. In joint performance, musicians closely monitor elements such as tuning and phrasing, actively listening to the effect of their own utterances and to one another’s. They make continuous, anticipatory adjustments, and–by actions such as breathing together, and through gestural responses to their partner’s direction of gaze–they demonstrate that they are attending to their co-performers [4,6]. The type of gestures that musicians are able to perform varies depending on the physical demands of holding and playing their instrument. Typical behaviours include upper body movements, upper torso (shoulder/neck/head) movements, nodding, tapping, expressive hand/arm gestures, facial cues including eyebrow-raises and sniffs, hip-swaying, and adapted conductor-like gestures which accommodate their instruments.

Musicians pursue the goal of producing integrated ensemble performances across various musical genres, all demanding different degrees of spontaneous invention on the part of the performer. Back-channeling has the most obvious utility for the more improvised forms of musical interaction. For example, jazz improvisation bears a close analogy to spontaneous conversation [11], with notions of ‘speaker’ and ‘listener’ commonly applied to such musical situations, while exploratory research has suggested that improvising North Indian classical musicians may use gestures with a social interaction (back-channel) function as a response to *being looked at* by a duo partner [6]. Nonetheless, improvised musical interaction differs from spontaneous conversation in its temporal dynamics. Unlike conversation, music is often characterised by explicit timing regularities that facilitate the entrainment, or coupling, of rhythmic behavior between individuals [12]. In rhythmically structured music, musicians play ‘together’ by timing their actions relative to hierarchically-arranged temporal frameworks centred on a regular underlying pulse [13]. Furthermore, while conversationalists take turn to speak, musicians often play concurrently, utilizing specialised cognitive-motor skills to anticipate, adapt, and attend to one another’s actions in real time [4]. Despite these potential differences, back-channeling cues may signal social contingency similarly in music performance and conversation, indicating mutual dependencies in the behaviour of self and other. In musical improvisation, such cues may function as a general, nonverbal mechanism for providing continuous feedback that facilitates musical beat entrainment, and also to enable co-performers to convey their attention to one another’s actions, which is essential for both the technical and expressive aspects of ensemble performance.

The current study was designed to examine whether back-channeling by musicians makes a contribution to third-party (observer) recognition of social contingency in musician dyads. Existing literature on musicians’ extra-musical behaviours offers various taxonomies and semiotic analyses of co-performer gestures, based on observational and ethnographic attention to both genre-specific conventions of physical gestures, and idiomatic behaviours related to particular instruments [7]. The current study differs in terms of its focus upon what we describe as the non-soloing musician, characterised as a contributor to the musical improvisation despite their temporary silence. In joint improvisation, musicians need not play continuously at the same time as one another and with equal prominence. As in natural conversation, there are likely to be moments (lasting from seconds to minutes) where one musician’s contribution is supportive rather than primary, and where they pull back or stop playing entirely while the other musician ‘solos’. What is the role of the non-soloing musician at such moments? They are still part of the duo and (in successful performances) likely remain a contributor to the improvisation both in their own estimation and also in the eyes of a third-party observer. In such instances, we use the expression *authentic social contingency* to describe the duo’s joint behaviour. In order to maintain unity, the behaviour of the non-soloing partner is likely to signal feelings, thoughts, and intentions both to their partner and to any observers. The non-soloing individual may demonstrate, for example, affective or evaluative evidence of appreciation for what the partner is doing (as in the case of the North Indian classical musicians described in [6]); their expert ‘insider’ understanding of some aspect of the musical solo being performed; and they may signal their readiness or otherwise for returning to play alongside the soloist, or to take a solo themselves. In this study, participants with varying levels of musical experience were presented with short audiovisual displays featuring excerpts of the improvised duo performances, showing moments where one performer plays (the soloist) and the other is temporarily silent, waiting to re-join within a matter of seconds (the back-channeler). As in work on observers’ identification of dyad affiliation in conversation [9,10], the range of back-channel cues was restricted to large-scale body movements–shown to be important in studies of musical communication [14]–by presenting point-light displays. Half of the displays showed ‘real’ duos from authentic episodes of musical interaction, while the other half showed ‘fake’ dyads, created by splicing together two members from separate duos. The task required participants to judge whether each display was real or fake.

We hypothesise that observers should be able to detect authentic back-channel cues within musical duos, and that this ability will be affected by the temporal dynamics of the interaction, the observer’s musical experience, and their rhythm perception skills. To address temporal dynamics, we compare two musical genres: standard jazz, which is based on a regular pulse, and free improvisation, in which a regular pulse is eschewed. Proponents of free improvisation have emphasised the structural and experiential similarities between free improvisation and non-musical social interaction [15]. Therefore, while a normal aptitude for everyday social interaction may provide enough awareness of joint communicative action for observers generally to perform well in judging free (non-pulsed) improvisations, this may not be the case for pulsed standard jazz. Indeed, recognizing authentic social contingency in standard jazz displays may be challenging, due the regular movements of soloist and back-channeler, which may encourage a bias to judge duos as real.

Musical experience may affect the ability to judge the authenticity of the dyad by influencing the degree to which the observer simulates the actions of soloing musician and back-channeler. It has been proposed that to understand the intention of interaction partners, an individual uses his or her motor system to simulate the other’s actions [16]. Brain imaging studies suggest that individuals most strongly simulate actions within their behavioural repertoire [17,18]. Therefore, if action simulation plays a role in perceiving back-channel cues, musical experience should have an influence on task-performance, with musicians outperforming non-musicians. Furthermore, experienced improvisers may show a specialism advantage, as observers familiar with playing in a particular musical style may be better at simulating actions for that style: jazz improvisers may be most sensitive to standard jazz displays, and free improvisers most sensitive to free improvisation displays.

Finally, the perception of back-channel cues may be influenced by the observer’s rhythm skills. Observers’ drumming expertise has been found to influence positively the accuracy of their judgements of audiovisual synchrony in point light displays of simple, regular drumming patterns [19]. Accordingly, high rhythm perception skill may be more strongly associated with high sensitivity to back-channeling authenticity when observing pulsed standard jazz than non-pulsed free improvisation.

## Method

### Design

The perceptual judgment experiment employed a 2 x 4 repeated measures design, with independent variables of Style (Standard, Free) and Group (Standard jazz musician; Free improviser musician; Non-improvising musician; Non-musician). Participants judged back-channel authenticity. The dependent measures were task sensitivity and response bias.

#### Duo recordings

Twenty-four improvising instrumental musicians were recruited and paired to form 12 duos. Six duos comprised of musicians who specialised in standard jazz; the other half comprised of specialist free improvisers. The musicians played a variety of instruments (see Table 1). All musicians were experienced in public improvised performance, typically for 10 or more years (free improvisers) or 7–10 years (standard jazz). Participants received financial compensation.

**Table 1:** Duo instrumentation and excerpt selection.

Instrument				10 s duo excerpts per instrument					
Duo		A	B	Viable	*Experimental trial*		*Practice trial*		*Not used*
					*Real*	*Fake*	*Real*	*Fake*	* *
**Free**	1	Flute	Double bass	5	* *	* *	* *	*1(A)*	*4*
	2	Drums	Soprano saxophone	0	* *	* *	* *	* *	* *
	3	Drums	Tenor saxophone	5	*2*	*1(A)*,*1(B)*	* *	*1(B)*	* *
	4	Alto saxophone	Clarinet	5	*2*	*1(A)*,*1(B)*	* *	*1(A)*	* *
	5	Soprano saxophone	‘Cello	5	*2*	*1(A)*,*1(B)*	* *	*1(B)*	* *
	6	Piano	Electric guitar	4	*2*	*1(A)*,*1(B)*	* *	* *	* *
**Standard**	7	Tenor saxophone	Piano	3	* *	* *	*1*	*1(A)*	*1*
	8	Electric guitar	Trumpet	5	*2*	*1(A)*,*1(B)*	* *	*1(B)*	* *
	9	Electric bass guitar	Tenor saxophone	4	*2*	*1(A)*,*1(B)*	* *	* *	* *
	10	Violin	Piano	4	*2*	*1(A)*,*1(B)*	* *	* *	* *
	11	Acoustic guitar	Double bass	4	*2*	*1(A)*,*1(B)*	* *	* *	* *
	12	Double bass	Piano	4	* *	* *	*2*	* *	*2*

The table shows number of viable excerpts retrieved (per instrument), and use of these excerpts as experimental stimuli and practice trials. Excerpts for Real duo stimuli involve both A and B instruments. Excerpts used to generate Fake duo stimuli use only one instrument (either A or B) combined with an instrument (either A or B) from a different excerpt.

#### Apparatus

Recordings were made using an optical motion capture system (Vicon, Oxford UK). Musicians wore 18 light-reflective markers: 4 on the head, 1 on the jugular notch between the clavicles, 1 on the sternum, and 1 on each shoulder, elbow, wrist, hip, knee, and ankle. Ten cameras positioned around the laboratory recorded the musicians’ body movements at 200Hz, using the Vicon Nexus 1.6.1 system to capture and model motion in three dimensions. Separate audio tracks were recorded for each musician, using two Audio Technica AT 2035 condenser microphones. Triggers were recorded on an audio track to allow offline synchronisation of audio recordings with motion-capture.

#### Material

The six duos in the Standard condition all played the same jazz standard, *Autumn Leaves* (J. Kosma, 1945), following a specified form:

- Main theme–duo
- Verse–duo
- Trading solos (taking turns to play solo sections, according to the verse’s harmonic structure)–solos (A, B, A, B)
- ‘Head’–duo

Although not as common as the ubiquitous ‘standards’ jam, free improvisation is a widely recognised form of music-making [20]. It is diverse in musical outcome by its very nature–but also relatively easy to constrain, as required for the purposes of this investigation. The six duos in the Free condition performed two- to three-minute free improvisations following the form:

- Player A leads, player B accompanies—duo
- A drops out, B plays unaccompanied solo–B solo
- A re-enters, back to duo—duo
- B drops out, A plays unaccompanied solo, A solo
- B re-enters, improvisation concludes—duo

Thus the instructions to the free improviser musicians were to take turns at playing solo and non-solo in a form designed to elicit attentive joint improvisation. The musicians were not hesitant, and did not express confusion regarding these instructions.

Each pair of musicians recorded an average of 11 takes.

#### Stimuli creation

**Fig 1:**
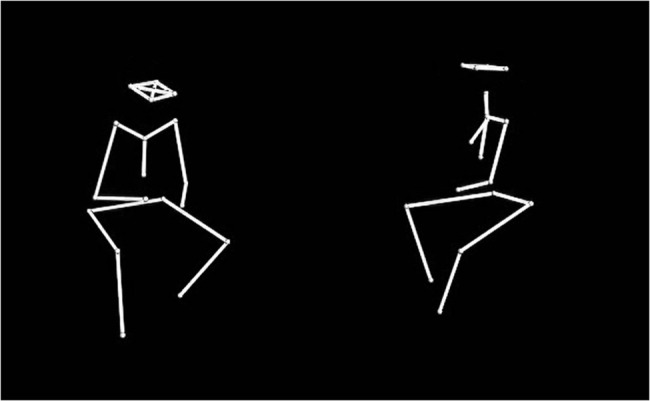
Still image taken from a video point-light display of a real musician duo. From left to right: Back-channeler, Trumpet; Soloist, Electric Guitar.

All solo/ non-soloist excerpts of at least 10 s duration were identified from the recordings. Quality criteria were applied, screening for missing markers and background noise. Using Windows Movie Maker, point-light representations were generated from the combined and synchronised Nexus (kinematic) data and audio recordings, displaying as white dots connected by white lines on a black background (Fig 1). Non-soloing (back-channeler) instrumentalists were positioned on the left-hand side of the screen; soloists on the right. These audiovisual displays were cut to 10 s.

The resulting set of animations included excerpts from 21 musicians from 11 of the 12 duos (see Table 2 for further details of excerpt usage). Excerpts from eight duos (four Free and four Standard) were used as the basis of 16 real and 16 fake duos (http://dx.doi.org/10.7488/ds/251.). The fake duos were generated in Windows Movie Maker, where original back-channelers were replaced with a back-channeler from a different duo (see Table 2), positioned to match the original back-channeler’s orientation toward the soloist. Nine of the remaining excerpts were used to generate six practice trials (three real and three fake).

**Table 2:** Instrumental pairings.

			Real		Fake	
	*Soloist*		*Non-soloist*		*Non-soloist*	
**Free**	Duo #3	A	Duo #3	B	Duo #5	B
	Duo #3	B	Duo #3	A	Duo #5	A
	Duo #4	A	Duo #4	B	Duo #6	B
	Duo #4	B	Duo #4	A	Duo #6	A
	Duo #5	A	Duo #5	B	Duo #3	B
	Duo #5	B	Duo #5	A	Duo #3	A
	Duo #6	A	Duo #6	B	Duo #4	B
	Duo #6	B	Duo #6	A	Duo #4	A
**Standard**	Duo #8	A	Duo #8	B	Duo #9	B
	Duo #8	B	Duo #8	A	Duo #9	A
	Duo #9	A	Duo #9	B	Duo #8	B
	Duo #9	B	Duo #9	A	Duo #8	A
	Duo #10	A	Duo #10	B	Duo #11	B
	Duo #10	B	Duo #10	A	Duo #11	A
	Duo #11	A	Duo #11	B	Duo #10	B
	Duo #11	B	Duo #11	A	Duo #10	A

Instrumental pairings of the authentic musician duos, as used to generate fake experimental trial stimuli. For example, the real Free duo pairings consisted of the original members (A and B) of duos #3, 4, 5 and 6; the fake Free duo pairings used excerpts featuring the same soloists from these duos, but added excerpts of non-soloist listening partners from duos #5,6,3 and 4 respectively. Musicians from duos #1,2,7 and 12 did not feature in the stimuli selection.

#### Extent of motion cues

Given the diversity of instrumentalists and individual styles of performance in the original recordings, the video excerpts were analysed to rule out differences in the overall extent of motion cues in the back-channeling musician as an explanation for differences in perceptual judgements. The mean quantity of motion (QoM) for each performer (back-channeler and soloist) in each stimulus video was calculated using VideoAnalysis software (http://www.uio.no/english/research/groups/fourms/software/VideoAnalysis/). Average QoM values (on a scale ranging from 0 [no pixel change from frame to frame] to 1 [all pixels change from frame to frame]) across conditions in back-channelers were as follows: Free real = .0166 (*SE* .0026), Free fake = .0168 (*SE* .0025), Standard real = .0192 (*SE* .0029), Standard fake = .0195 (*SE* .0030). Average QoM values for soloists were as follows: Free real = .0225 (*SE* .0013), Free fake = .0240 (*SE* .0013), Standard real = .0208 (*SE* .0018), Standard fake = .0209 (*SE* .0019). A Style (free vs. standard) x Authenticity (real vs. fake) x Instrumentalist (back-channeler vs. soloist) ANOVA did not yield any statistically significant main effects or interactions (p > .05), indicating that QoM did not vary as a function of Style or Authenticity, though there was a near-significant tendency for greater motion in soloists than back-channelers (F(1,14) = 3.85, p = .07). These findings suggests that despite the range of different instruments involved in each duo, there is no overall difference in the degree of visible motion related to back-channel cues either between Free and Standard excerpts, or indeed when comparing real versus fake excerpts.

### Perceptual judgement task

#### Participants

A sample of 60 participants was recruited for the perceptual judgment task. Sample size was determined via an a priori power analysis (using GPower [21]) based on effect sizes from previous studies of the ability to detect cues to social interaction [9,22] and expressive intentions in movement kinematics [23].

The sample included four groups of participants

- **Jazz Improvisers.** 15 musicians specialising in jazz improvisation. Mean age 35.9 years (SD 17.0); 20.2 years ensemble experience (SD 15.0). 12 male.
- **Free Improvisers.** 15 musicians specialising in free improvisation. Mean age 36.9 years (SD 5.7); 15.5 years ensemble experience (SD 8.9). 11 male.
- **Classical musicians.** 15 classically-trained (non-improviser) musicians: Mean age 25 years (SD 3.6); 8.8 years ensemble experience (SD 6.9); 5 male.
- **No musical training.** 15 non-performers with no instrumental training. Mean age 25.8 years (SD 4.1); 6 male.

#### Ethics statement

Participants were paid for their time and travel. The study received written approval by ethics committees at the University of Leipzig and the University of Edinburgh.

#### Procedure

Participants were tested individually with a computer running ‘Presentation’ software (www.neurobs.com). Participants were instructed that they would see duos performing musical improvisations together, but not that there were two types of improvisation. Given the unfamiliarity of the free improvisation style to many people, they were advised that while some excerpts might sound unusual that they should do their best to complete the task regardless.

Each trial presented a 10-second point-light audiovisual movie clip, after which participants were asked to identify whether the duo was real or fake. Responses were made using two labelled keys on the computer keyboard. Trials were initiated by pressing the spacebar. Participants completed six randomly-presented practice trials, during which they were informed whether each response had been correct or incorrect. This was followed by four blocks of 32 experimental trials without feedback. Each block contained 16 real and 16 fake clips, which were repeated across blocks with presentation order randomised.

At the end of the experiment, rhythm perception skills were measured via the rhythm subtest of the Musical Ear Test [24]. Participants also completed a questionnaire assessing musical background and task strategy. The experiment took approximately 1 hr 30 min.

## Results

### Sensitivity to back-channeling authenticity

**Fig 2:**
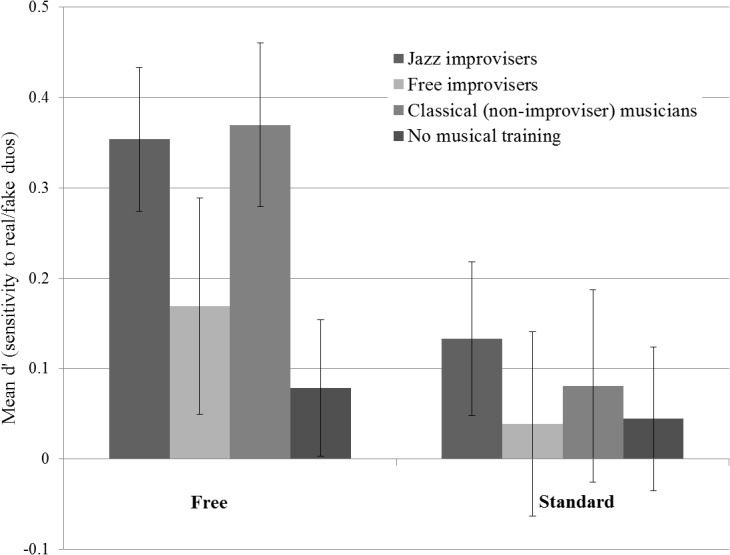
Mean d’ (sensitivity) for all four participant groups in the two Style conditions (Free and Standard improvisation). Vertical axis represents participants’ sensitivity to the real/fake identification task (0 = insensitive, 1 = most sensitive). Error bars show standard error.

Participants’ sensitivity to the real/fake distinction was assessed by computing d’ [25]. Average d’ in each of the two improvisation style conditions for each of the four groups is displayed in Fig 2. A Style x Group ANOVA on these data yielded a significant main effect of Style (F(1,56) = 5.90, p < .05), while the main effect of Group and the Style*Group interaction were non-significant (p > .05). One sample t-tests on the full sample revealed that participants were sensitive to the real/fake distinction in the Free condition (t(59) = 5.05, p < .001) but not in the Standard condition (http://dx.doi.org/10.7488/ds/251).

Following the significant main effect of Style, data from Free and Standard conditions were entered into separate regression analyses to test the hypothesis that rhythm skill is predictive of sensitivity to back-channeling authenticity. In the Free condition, the model is not significant (R^2^ = .015, F(1,58) = 0.89, p = n.s), suggesting that individual differences in real/fake judgments are not related to rhythm perception skill. In the Standard condition, however, the model is significant, showing that performance in the rhythm task accounts for 11% of the variance in d’ results (R^2^ = .11, F(1,58) = 7.18, p = .01). Therefore, while the participant sample overall did not reliability judge real from fake Standard duos, those individuals with good rhythm perception skills were most likely to do so.

### Response bias

**Fig 3:**
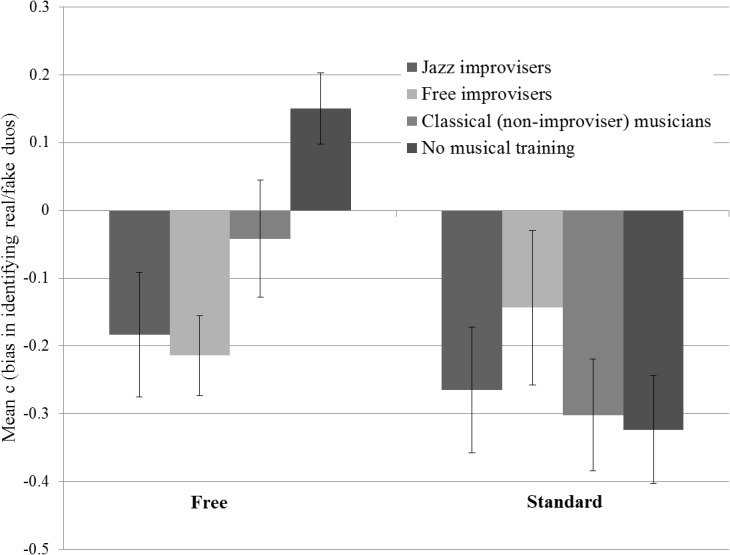
Mean C (bias) for all four participant groups in the two Style conditions (Free and Standard improvisation). Scores below zero indicate a bias to judge items as real, while scores above zero indicate a bias to judge items as fake.

Biases to respond that the duos were either real or fake independently from true duo authenticity were assessed by computing c scores [25]. The average c score in each of the two Style conditions for each of the four Groups is shown in Fig 3. The ANOVA for these data yielded a significant main effect of Style (F(1,56) = 9.996, p < .01), indicating a general bias to judge Standard improvisation items as real. The main effect of Group was not significant, but there was a significant Style*Group interaction (F(3,56) = 3.969, p = .01).

To explore Group differences in the Free condition, planned contrasts revealed a significant effect of musical experience between musicians and nonmusicians (t(36.2) = -4.24 (not assuming equal variances), Cohen’s d = -1.41, R^2^ = .34, p < .001). While nonmusicians exhibited a bias to report free improvisations to be fake irrespective of their true class of authenticity, musicians ranged from showing no bias, to showing a bias towards reporting free improvisations as real. However, there was no significant difference between improvising and non-improvising musicians’ task performance, and no specialism advantage for improvisers in their style. In the Standard condition, the planned contrasts revealed no significant effect of musical experience on bias.

Separate regression analyses were conducted to ascertain whether response biases in the Free and Standard conditions were related to rhythm perception skill. For the Free condition, rhythm skill is a significant predictor of response bias (R^2^ = .11, F(1,58) = 7.10, p = .01). Individual differences in rhythm perception skill therefore accounted for 11% of variation in response bias, with individuals with high rhythm perception skills showing a bias to judge Free improvisations as real. For the Standard condition, the regression model is non-significant (R^2^ = .00, F(1,58) = .01, p = ns).

## Discussion

The current study investigated the perception of nonverbal back-channeling cues associated with the body movements of musicians engaged in turn taking during standard jazz and freely improvised performances. Our main finding is that individuals with different levels of musical experience (standard jazz musicians, free improvising musicians, non-musicians, non-improvising musician) were able to detect back-channel cues in freely improvised musical interactions. This finding supports our main hypothesis that back-channeling signals social contingency in freely timed musical interactions, analogously to effects observed in the context of spoken conversation [9,10].

We suggest that the similarity between spontaneous conversation and free improvisation allowed lay observers to use their aptitude for everyday social interaction to form judgements of the musical stimuli. Our measure of rhythm perception skill did not explain sensitivity variation in the Free condition, and neither did we find a sensitivity main effect at the Group-level differentiation of musical experience.

However, in the free improvisation condition we found a significant difference between musicians and non-musicians regarding bias: while non-musicians tended to report free improvisation duos as fake, musicians ranged from no bias to a bias towards real. This partially supports our hypothesis that musicians would outperform non-musicians, as they show weaker bias in response. Musicians’ learned skill in generating an internal referent pulse may facilitate ‘top-down’ imposition of rhythmic structure, offering enhanced receptiveness to non-obvious inter-performer synchrony. It is also plausible that musically-untrained participants are less familiar with the concept of freely improvised performance, and more likely to experience a conflict between the assumption that ensemble performance should entail rhythmic, interpersonal synchrony, and the fact that freely timed solos do not obviously afford such synchrony. However, we found no evidence for enhanced sensitivity to the back-channeling by improvisers over non-improvisers, nor by specialist improvisers observing duo performance of their own genre. Therefore we suggest that the perception of back-channeling in free interactions is a general social ability.

In the Standard condition, participants as a whole sample did not distinguish reliably between real and fake duos; however, rhythm perception skill was–as hypothesized–found to explain some variance in sensitivity. We also found a bias for all participants to report duos as real in the Standard condition. This may be due to the greater homogeneity of the Standard duo performances, compared to Free. While timing decisions were left to the performers in every case, all Standard excerpts have similar tempi of 120–150 beats per minute, relating to a regular interval of approximately 400–500 ms. Audio-visual simultaneity is reported to be perceived within a 200 ms integration window [26]. Given the similarity of the Standard performances, such a range of acceptable asynchrony makes the distinction between true entrainment (characterised by coupling and interaction) and apparent synchronization (two processes unfolding at the same rate but independently) perceptually challenging. Nonetheless, the significance of rhythm test scores in explanation of sensitivity variance could suggest that for participants with the highest temporal acuity, the audio-visual integration window becomes narrower or that these participants are better able to perceive rhythmic regularities in the body movements of the displayed soloists and their back-channeling partners.

While task performance in terms of d’ shows that observers in general could discriminate between authentic and mismatched Free duos, the overall performance was fairly weak. The closest existing research for comparison is an agency identification study [23] where participants were asked to identify point-light representations of their own expressive actions versus those of another person. The average d’ scores (~2.5) were an order of magnitude higher than the present study (~.25). The apparent difference in performance across these studies may be attributable to differences between the aims and methods. In contrast to our study, the authors [23] employed a design in which the same participants who were recorded dancing returned after a period of several months to perform various recognition tasks based on the point-light animations. However, participants were not asked to identify real versus fake dancing dyads but asked to judge whether the point-light animation of an individual dancing expressively represented themselves or whether it represented another person. Our task was considerably harder, given both the minimal nature of the visual presentation and the unfamiliarity for many participants with freely improvised musical performance.

Evidence that observers perceive the real/fake distinction based solely on musical sound and body movement despite the difficulty of the task is an important finding. Future studies could explore conditions that lead to improved judgment accuracy. For example, presenting the two genres of Free and Standard improvisation separately in a blocked design could lead to improved task performance by giving observers longer to ‘tune in’ to nuances of the ostensive musical communication.

It is also possible that musicians may make more accurate judgements when observing performances on instruments that they themselves played [27]. A larger scale study would need to recruit more widely to find musician participants who could meet the criteria of being expert improvisers paired into duos on matched instruments across musical genre conditions. Related to this point, one might also take into consideration the typical movement or explicit cueing behaviours of various instrumentalists–for example, pianists’ hands are typically hidden, while trumpeters or guitarists cannot easily disguise their intention to begin playing. However, the quantity of motion analysis ruled out the extent of visible motion as a causative factor in participants' judgments in the two styles of Free and Standard improvisation. Further research would be required to offer a fuller account of both stylistic or genre-based norms and instrument-specific gestures.

We can yet speculate about the strategies that participants used to fulfil the task. In a post-test questionnaire, participants reported the strength to which they had relied on various cues in making their judgement about the authenticity of the duo, including musical ‘beat’, head movement, body movement, upper body movement, arms and legs. Success at the task as indicated by d’ was found to be correlated (positively) only with attention to musical ‘beat’, and only in the Standard condition (r(58) = 0.292, p = 0.023). This result is consistent with the finding that greater rhythm acuity was associated with sensitivity to the task in the Standard condition.

## Conclusion

The results of the current study suggest that the detection of authentic back-channeling cues in musical duo improvisation depends on whether the musical interaction is rhythmically regular or freely timed, and, if regular, on the observer’s rhythm perception skills. The finding that individuals were generally able to distinguish between real and fake displays of free improvisation suggests that sensitivity to musical back-channeling cues in conversation-like episodes of freely timed musical interaction may be a general ability that is independent from musical specialisation and rhythm skills. Sensitivity to back-channeling in rhythmically pulsed standard jazz improvisations, on the other hand, was found to depend on the specific musical skill of rhythm perception.

Although musical background was not found to affect sensitivity to back-channel cues, it was found to bias authenticity judgments. Non-performers, without any musical training, tended to judge free improvisation duos as fake. While some musically-experienced observers exhibited little to no bias, others had a tendency to judge free improvisation duos as real. This tendency was correlated positively with rhythm perception skill, a result for which we offer the explanation that proficiency at generating an internal pulse may have enabled rhythmic structure to be imposed–rightly or wrongly–on the free improvisations in a top-down fashion.

The experimental paradigm we employed, involving only audio and movement kinematics, demonstrated that musical duo identification is possible even in impoverished stimuli. The findings demonstrate that the social integration of two musicians into a single, communicating dyad is an aspect of musical performance to which observers are sensitive. These results emphasise the functional role of the listening non-soloist (back-channeler) in specialist musical dyadic interaction, and suggest that observer perception of this role may depend upon general social abilities. The findings invite further study of links between the interpersonal dynamics in conversation and in musical improvisation.
